# Chemical biology of genomic DNA: minimizing PCR bias†

**DOI:** 10.1039/c4cc05107f

**Published:** 2014-10-18

**Authors:** Gordon R. McInroy, Eun-Ang Raiber, Shankar Balasubramanian

**Affiliations:** Department of Chemistry, University of Cambridge, Lensfield Road, Cambridge, CB2 1EW, UK

## Abstract

The exquisite selectivity of chemical reactions enables the study of rare DNA bases. However, chemical modification of the genome can affect downstream analysis. We report a PCR bias caused by such modification, and exemplify a solution with the synthesis and characterization of a cleavable aldehyde-reactive biotinylation probe.

The application of chemical tools for the study of biological questions is a key tenet of chemical biology. The importance of such approaches to genomic studies has grown with the utilization of chemically reactive small molecule probes for the mapping of modified bases in the genome. Such methods have exploited both the intrinsic chemical reactivity of certain modifications, and new functionalities introduced by chemical treatment of nucleic acids, to install detectable and capturable tags.

Methylation at the C5 position of cytosine is an established epigenetic mark, playing an essential role in processes such as X-chromosome inactivation, transcriptional regulation^1^ and transposon silencing.^2^ Its TET-mediated iterative oxidation products, 5-hydroxymethylcytosine (5hmC), 5-formylcytosine (5fC) and 5-carboxycytosine (5caC), are also present in low abundance in mammalian genomes. Though less well-understood than 5-methylcytosine (5mC), they have now been implicated in active DNA demethylation^3^ and epigenetic processes.^4^ Central to the growing understanding of these DNA base modifications has been the development of techniques for their genomic mapping, many of which have involved chemical biology approaches. Enrichment methods for genomic mapping of the marks have been widely used, with the majority of approaches taken employing chemical modification of DNA in the workflow. Such methods involve isolating fragments of the genome that contain a particular mark, followed by sequencing to reveal where in the genome it can be found. The chemical methods have exploited the particular functional groups of 5hmC,^5^–^7^ 5fC,^8^,^9^ and 5caC,^10^ to install a capturable tag such as biotin. This allows for a selective enrichment of tagged fragments with streptavidin-coated beads, before sequencing and bioinformatics analysis.

Due to the low levels at which the modifications are present, for example, there are approximately ten 5fC per million total cytosine species in a mammalian genome,^11^ post-enrichment PCR amplification is customarily required before DNA sequencing is possible. PCR amplification does not result in an equal distribution of fragments; indeed libraries generated using PCR are known to have a biased composition.^12^ Whilst this effect is often due to the sequence composition, for example GC rich sequences are known to be underrepresented following PCR amplification, polymerase stalling at unnatural base modifications can be another source of bias.^13^ Indeed it has been shown that bisulfite treated 5hmC forms an adduct, 5-methylenesulfonate, which can cause polymerase stalling and thus lead to underrepresentation of densely hydroxymethylated regions.^14^ Several methods previously mentioned leave significant chemical scarring on DNA (Fig. 1), resulting from either the entire probe being left in place or some significant chemical residue following cleavage. It is therefore important to consider whether PCR amplification leads to an underrepresentation of fragments containing the modification of interest in the final library. Considering the scarcity of these modifications, this may lead to peaks being lost in background noise. Herein we describe a PCR bias caused by a commercial aldehyde reactive probe, and report the synthesis of a cleavable version that leaves minimal chemical scarring following cleavage and resolves the bias.

We synthesised a probe† (Scheme 1A) capable of installing a biotin moiety at aldehyde containing sites, as found in 5fC or generated from the periodate cleavage of vicinal diols such as glucose.^5^ Probes with such chemical reactivity have been employed in recently developed techniques for mapping 5hmC^5^ and 5fC.^8^ Additionally we introduced a cleavage site at the reactive terminus to allow a near complete scission of the probe from its substrate. For this we utilized an azide masked hemiaminal ether, which may be cleaved by Staudinger reduction, and the subsequent decomposition of the highly unstable hemiaminal ether (Scheme 1B). The moiety is stable to heat, pH and oxidative conditions, and yet under mild, reductive and non-denaturing conditions quantitative cleavage occurs. This cleavage chemistry is key to Solexa/Illumina DNA sequencing, where its biocompatibility and cleavage efficiency are clearly demonstrated.^15^ Our probe was synthesised in five steps from 2-bromomethyl-1,3-dioxolane. Initially, the cleavage site was formed by treatment with trimethylsilyl azide in the presence of a Lewis acid. The reactive hydroxylamine moiety was then installed with mesylation activation followed by an *O*-alkylation of boc-protecting hydroxylamine to give **4**. Alkaline ester hydrolysis and pentafluorophenyl (Pfp) activation allowed coupling with ethylamine modified biotin to introduce the capture tag. A final boc-deprotection with methanolic HCl and RP-HPLC purification yielded the pure probe **1**.

The aldehyde reactivity and selectivity of the probe was confirmed by incubating 15-mer oligonucleotides† containing cytosine, 5mC, 5hmC or 5fC with **1** in the presence of *p*-anisidine at pH 5. Analysis using LC-MS revealed a DNA-probe adduct formed only between the 5fC-containing DNA and **1**, with the mass expected following oxime formation at the 5fC aldehyde (Fig. 2). Additionally, the efficiency of cleavage induced by azide reduction was evaluated. We found tris-(2-carboxyethyl)phosphine (TCEP) buffered with Tris-HCl pH 7.4 to be capable of facilitating the quantitative conversion of the DNA-probe adduct to 5fC-oxime (5foxC) containing DNA within 15 min at 65 °C.

To measure the effect of chemical tags on amplification efficiency, quantitative real-time PCR (qPCR) was employed. 100-mer oligonucleotides containing one, four or ten 5fC sites were generated by PCR and reacted with either a commercially available aldehyde reactive probe^16^ (ARP) or probe **1**. Additionally, samples of DNA-1 adducts were further treated with TCEP to yield DNA containing the 5foxC cleavage product. The threshold cycle (Ct) obtained for 1 pg of DNA input was normalised against a 5fC control to give a ΔCt value indicative of the modification induced PCR bias, where higher values represent a decrease in amplification efficiency. Samples treated with either ARP or **1** led to similar ΔCt profiles, where low adduct numbers led to a delay of one cycle, while higher modification density caused a significantly greater change of over two cycles (Fig. 3). In contrast, the strands containing the 5foxC cleavage product induced no significant ΔCt from the control template, irrespective of the modification density, and greatly reduced the density dependent PCR inhibition effect. These findings show that DNA scarring, in this case due to the presence of a capturable biotin tag, may lead to underrepresentation following inefficient PCR amplification. This matter may be resolved by the strategic placement of a cleavage site to minimize chemical scarring.

To further investigate the apparent chemical scarring induced PCR bias, we performed polymerase primer extension reactions with differentially modified synthetic oligomer substrates. The substrates were 100-mers with five modification sites between positions fifty and sixty. Single primer extension amplification was performed using fluorescently labelled primers, with aliquots taken after each cycle and run on a high resolution denaturing polyacrylamide gel. The multi-cycle approach allowed the visualisation of minor pause sites that would not be apparent after a single primer extension due to low intensity.

The unmodified template showed only product bands (Fig. 4B-a), which confirmed there were no inherent sequence or secondary structure induced pause sites. Likewise, the 5fC containing template was amplified effectively (Fig. 4B-b), though the presence of very faint bands suggests 5fC may have some effect on polymerase processivity or DNA structure. However, the small-molecule probe containing the template resulted in strong polymerase pause sites (Fig. 4B-c), with two distinct truncation products evidently coinciding with the modified region. The pause sites suggest that the polymerase encounters difficulty in translocating the bulky base adducts into or through the active site.^17^ When probe **1** was cleaved from the template to yield 5fC-oxime containing DNA, polymerase stalling was clearly diminished (Fig. 4B-d), indicating that the near-natural base generated following chemical cleavage is not a major obstacle to PCR. Multiple extensions allowed visualisation of faint pause sites, suggesting that even the minimal residue could inhibit polymerase replication to some extent. The 5fC-oxime residue adds little steric bulk, but introduces additional hydrogen bonding possibilities and alters the tautomerization equilibrium,^18^ which may affect the DNA structure or polymerase processivity.

In conclusion, we have shown that the presence of chemical scarring following DNA labelling can cause DNA polymerase inhibition. The severity of this inhibition is related to the increasing modification density. Additionally, we have synthesised an aldehyde reactive biotin probe that may be removed under mild, nucleic acid compatible conditions to leave a minimal chemical residue. Cleavage of this probe from a DNA substrate resulted in abolition of the PCR bias effect. The application of chemical tagging approaches to genomic questions has yielded powerful methods, and will certainly continue to feature in our study of the genome. However, we must not neglect the effects on downstream processes such as PCR amplification and sequencing in our experimental design and analysis.

## Supplementary Material

† Electronic supplementary information (ESI) available: Synthetic procedures, molecular characterisation and oligonucleotide sequences. See DOI: 10.1039/c4cc05107f

ESI

## Figures and Tables

**Fig. 1:**
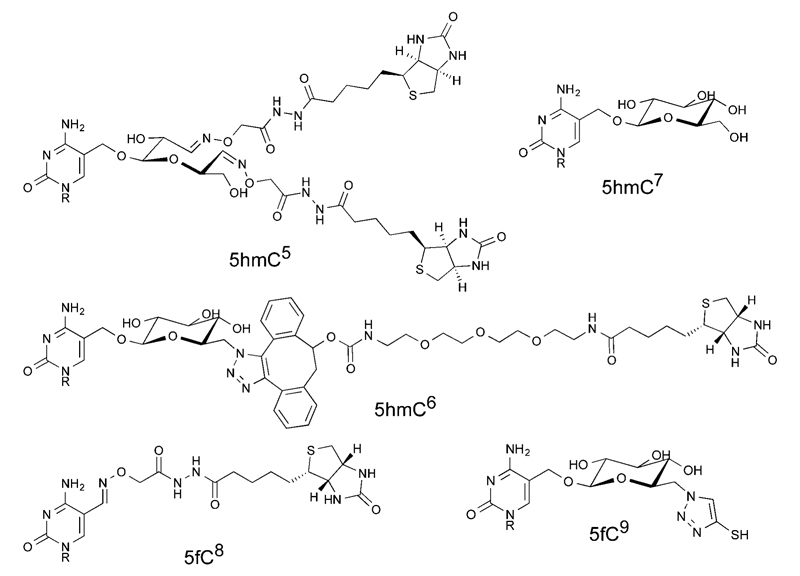
Structures describing examples of molecular scarring resulting from DNA probe interactions. Structures show increased steric bulk and unnatural functional groups. Each structure is labelled with the target modification and a literature reference.

**Fig. 2:**
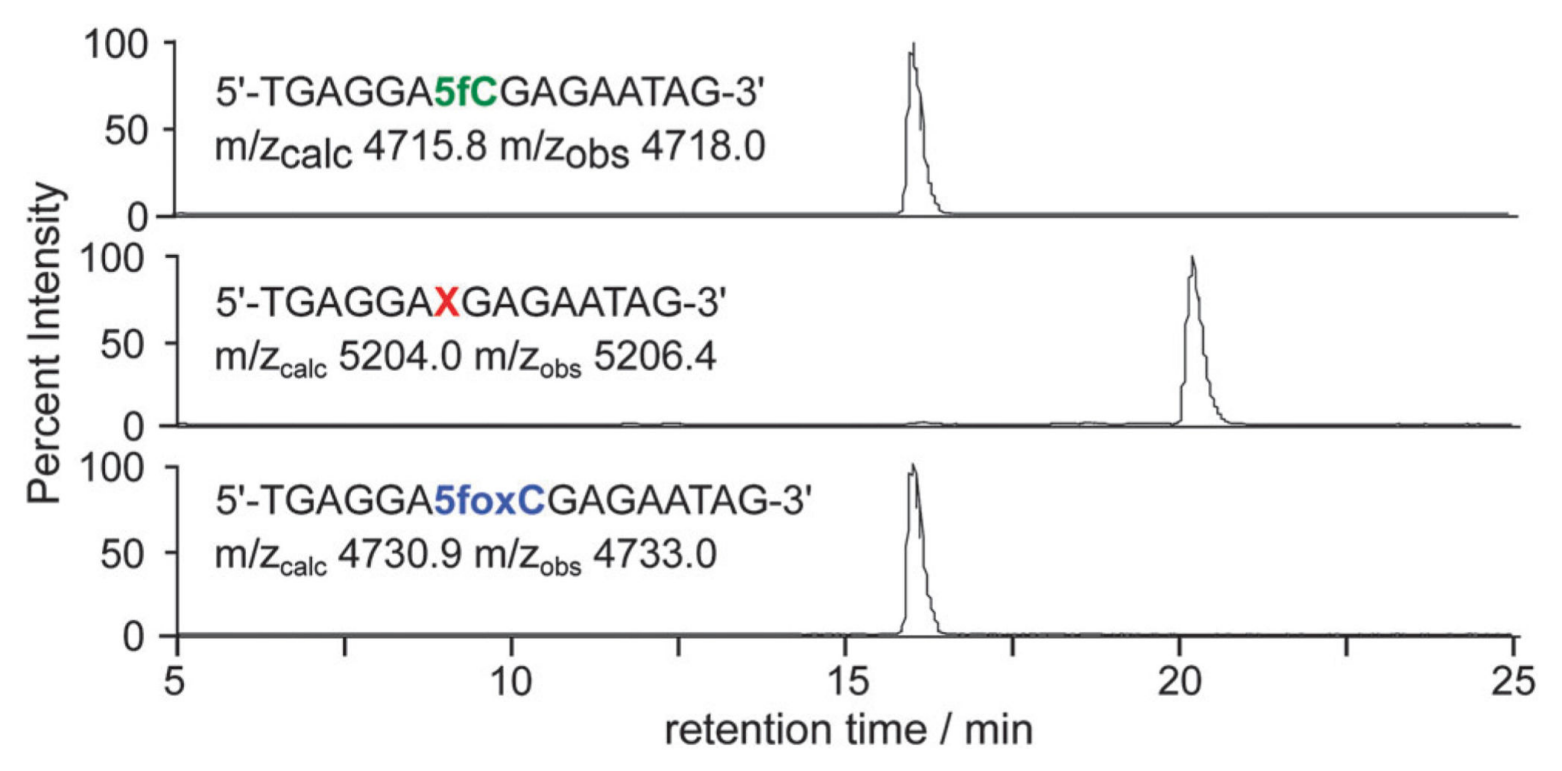
LC-MS was used to follow 5fC-containing DNA (top) through incubation with probe **1** at 37 °C for 24 h (middle), and subsequent probe cleavage with TCEP at 65 °C for 15 min (bottom). Base X indicates a 5fC–**1** adduct; traces are base peak chromatograms.

**Fig. 3:**
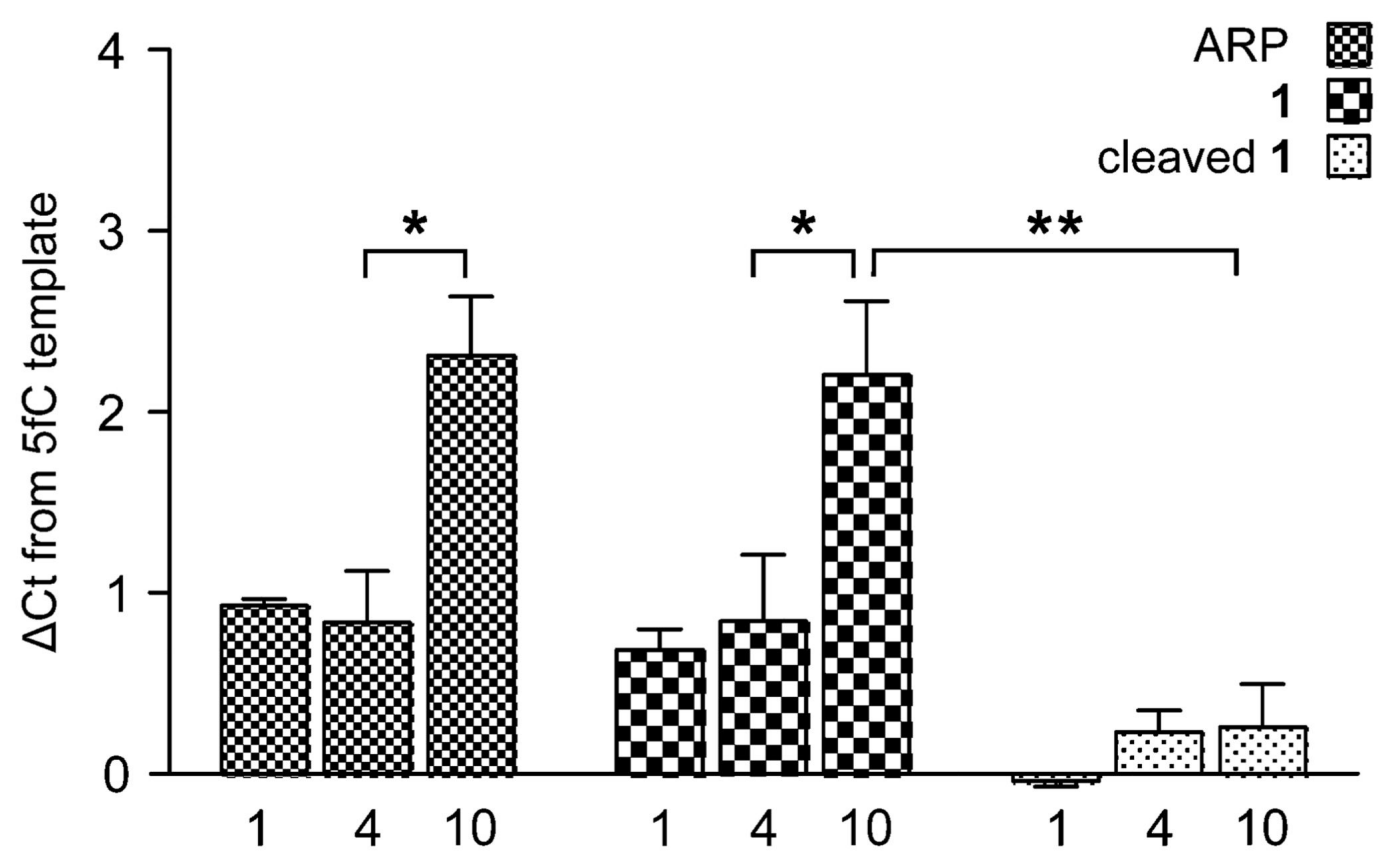
A qPCR study showing the relationship between DNA-adduct character, prevalence, and inhibition of PCR. ΔCt is relative to a 5fC-containing template. Bars show the average of three experiments, each performed in technical triplicate; error bars show the S.E.M. Statistical significance was calculated using two-way ANOVA (*P < 0.001, **P < 0.0001).

**Fig. 4:**
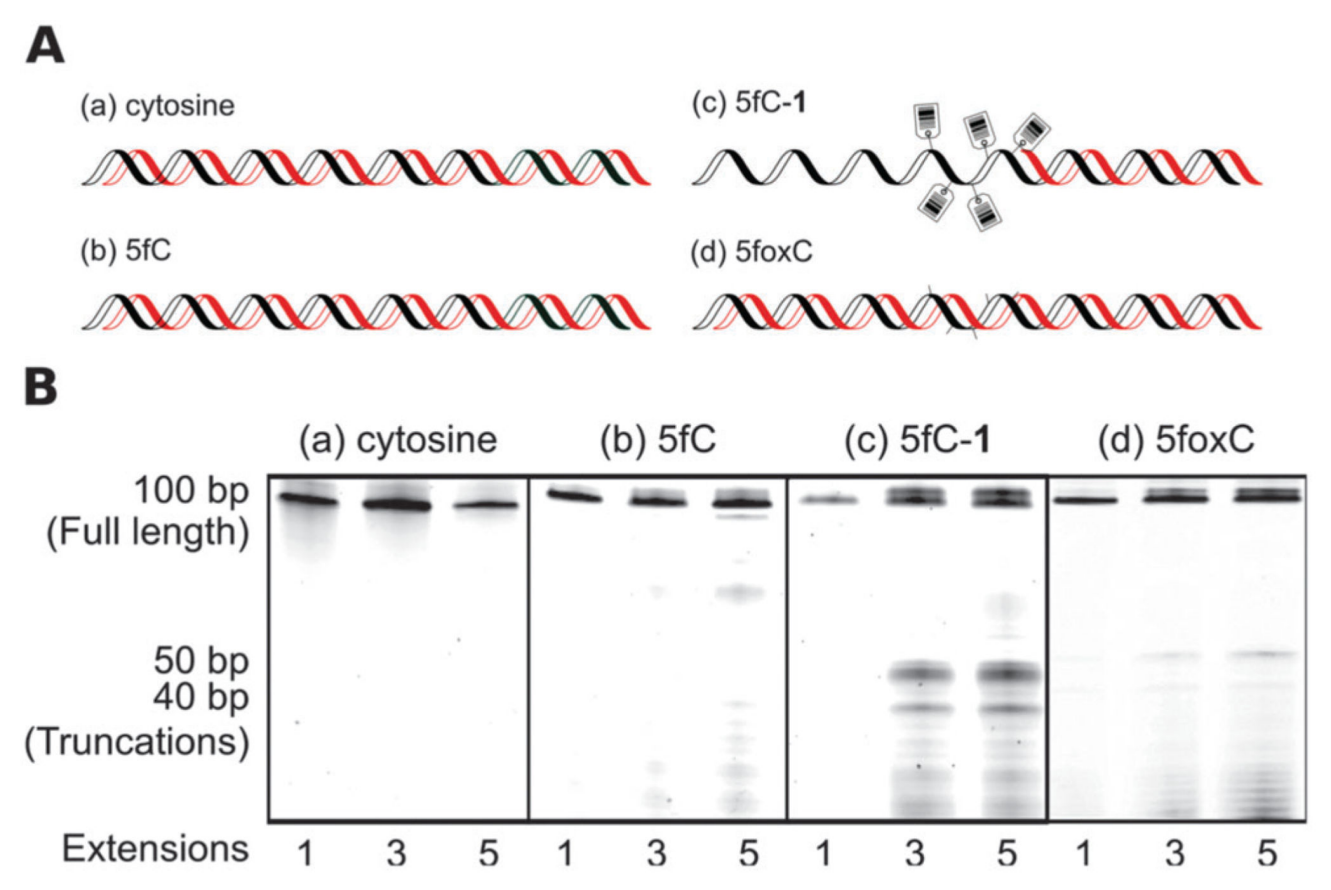
Templates containing five modification sites between positions fifty and sixty were templates for a primer extension assay: (a) no modification (b) 5fC (c) DNA-**1** or (d) DNA-**1** cleavage products. (A) Schematic showing primer extension along a template (black) to give a product (red). Chemical tagging inhibits polymerase action and yields truncated products; probe cleavage rescues this effect. (B) Denaturing PAGE shows probe-induced truncation products at modified sites, and the rescue achieved after chemical cleavage to yield near-natural DNA.

**Scheme 1:**
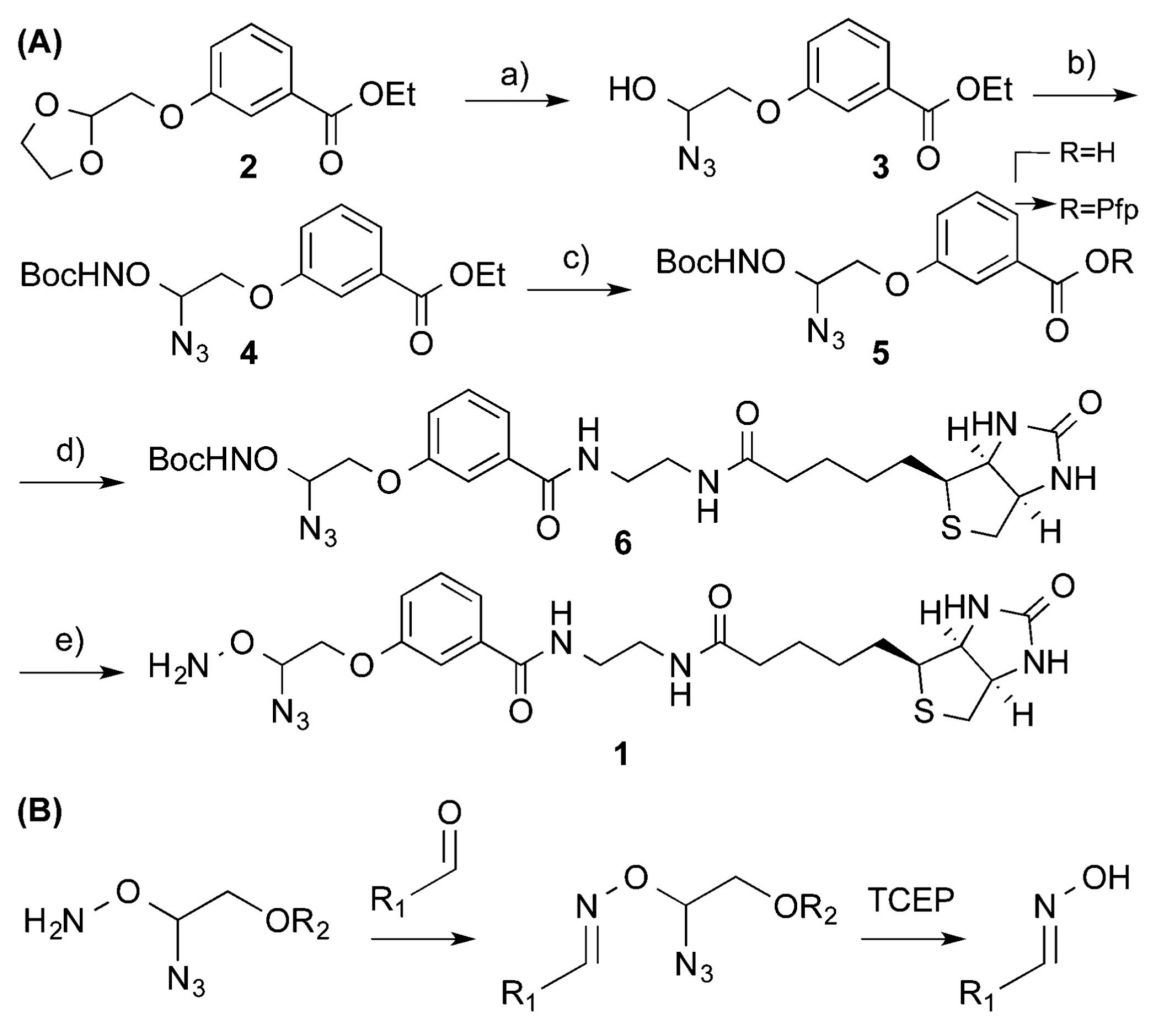
(A) Synthesis of cleavable probe **1** (a) TMS-N_3_, SnCl_4_ (b) (i) MsCl, Et_3_N, DCM (ii) BocNHOH, DBU, Et_2_O (c) (i) NaOH, EtOH (ii) PfpTFA, Et_3_N, Et_2_O. (d) Biotin ethylenediamine, DMF (e) HCl:MeOH. (B) Reaction of **1** with the aldehyde moiety and subsequent cleavage to yield the oxime.

## References

[1] Deaton AM, Bird A (2011). Genes Dev.

[2] Suzuki S, Ono R, Narita T, Pask AJ, Shaw G, Wang C, Kohda T, Alsop AE, Marshall Graves JA, Kohara Y, Ishino F (2007). PLoS Genet.

[3] Kohli RM, Zhang Y (2013). Nature.

[4] Wossidlo M, Nakamura T, Lepikhov K, Marques CJ, Zakhartchenko V, Boiani M, Arand J, Nakano T, Reik W, Walter J (2011). Nat Commun.

[5] Pastor WA, Huang Y, Henderson HR, Agarwal S, Rao A (2012). Nat Protoc.

[6] Song C-X, Szulwach KE, Fu Y, Dai Q, Yi C, Li X, Li Y, Chen C-H, Zhang W, Jian X, Wang J (2011). Nat Biotechnol.

[7] Yu M, Hon GC, Szulwach KE, Song C-X, Jin P, Ren B, He C (2012). Nat Protoc.

[8] Raiber E-A, Beraldi D, Ficz G, Burgess HE, Branco MR, Murat P, Oxley D, Booth MJ, Reik W, Balasubramanian S (2012). Genome Biol.

[9] Song C-X, Szulwach KE, Dai Q, Fu Y, Mao S-Q, Lin L, Street C, Li Y, Poidevin M, Wu H, Gao J (2013). Cell.

[10] Lu X, Song C, Szulwach K, Wang Z, Weidenbacher P, Jin P, He C (2013). J Am Chem Soc.

[11] Ito S, Shen L, Dai Q, Wu SC, Collins LB, Swenberg JA, He C, Zhang Y (2011). Science.

[12] Aird D, Ross MG, Chen W-S, Danielsson M, Fennell T, Russ C, Jaffe DB, Nusbaum C, Gnirke A (2011). Genome Biol.

[13] Aller P, Rould MA, Hogg M, Wallace SS, Doublié S (2007). Proc Natl Acad Sci U S A.

[14] Huang Y, Pastor WA, Shen Y, Tahiliani M, Liu DR, Rao A (2010). PLoS One.

[15] Bentley DR (2008). Nature.

[16] Kubo K, Ide H, Wallace SS, Kow YW (1992). Biochemistry.

[17] Kirouac K, Basu A, Ling H (2013). J Mol Biol.

[18] Hashimoto H, Hong S, Bhagwat AS, Zhang X, Cheng X (2012). Nucleic Acids Res.

