# Supplementary material for: Chemical biology of genomic DNA: minimizing PCR bias†
Source: Chem Commun (Camb). Author manuscript; Available in PMC 2018 Jan 9. (PMC5759930; doi:10.1039/c4cc05107f)
Supplement: ESI [file NIHMS75582-supplement-ESI.pdf]

Electronic Supplementary Material (ESI) for ChemComm.
This journal is © The Royal Society of Chemistry 2014

Chemical Biology on Genomic DNA: minimizing PCR bias

Gordon R. McInroy, Eun-Ang Raiber, & Shankar Balasubramanian

Department of Chemistry, University of Cambridge, Lensfield Road, Cambridge, CB2 1EW, UK

Electronic Supplementary Information (ESI) for Chemical Communications

Oligomer sequences for LC-MS analysis

15_C
`5′-TGA GGA CGA GAA TAG-3′`

15_5mC
`5′-TGA GGA (5mC)GA GAA TAG-3′`

15_5hmC
`5′-TGA GGA (5hmC)GA GAA TAG-3′`

15_5fC
`5′-TGA GGA (5fC)GA GAA TAG-3′`

Reaction conditions

15_5fC-1

We incubated the 15_5fC oligomer with compound **1** (400 uM and *p*-anisidine (100 mM) in NH_4OAc (40 mM) at 37 ^{o}C for 24 hours. We purified the oligomer by Mini Quick Spin Oligo Columns (Roche).

15_5foxC

We incubated thee 15_5fC-**1** oligomer with tris(2-carboxyethyl)phosphine pH 7.4 (100 mM) in Tris-HCl pH 7.4 (100 mM) at 65 ^{o}C for 15 minutes. We purified the oligomer by Mini Quick Spin Oligo Columns (Roche).

LCMS Chromatograms

Oligos were obtained HPLC purified from Eurogentec. LC-MS was performed on a Bruker amaZon system, with XTerra MS C18 Column, 2.5 µm, 2.1 x 50 mm. Solvents were A (10 mM TEA, 100 mM HFIP) and B (MeOH), and a gradient from 5 % B increasing at 1 % per minute.

15_5fC

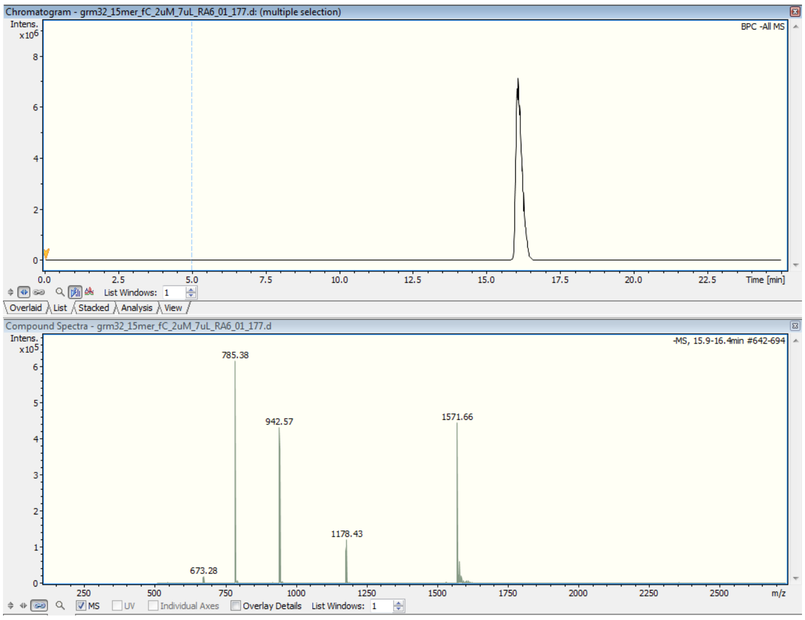

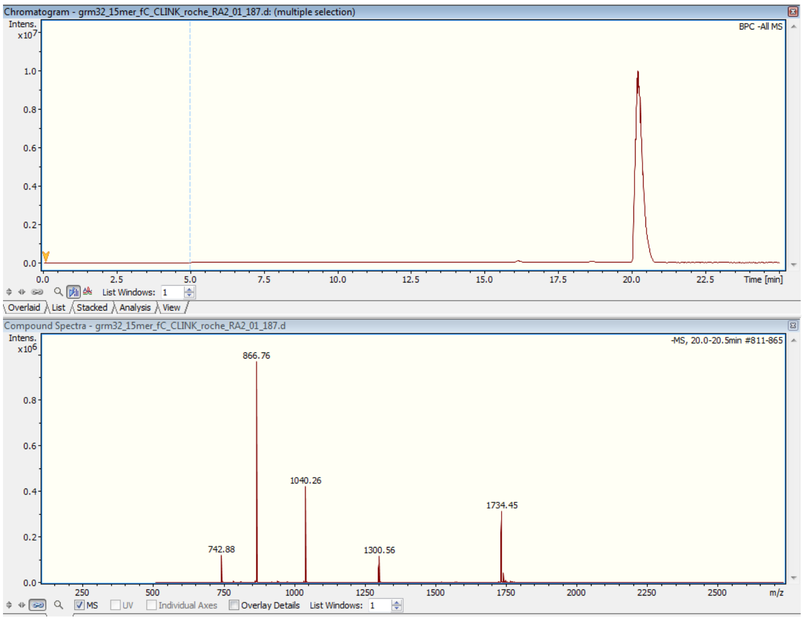

15_5fC-compound1

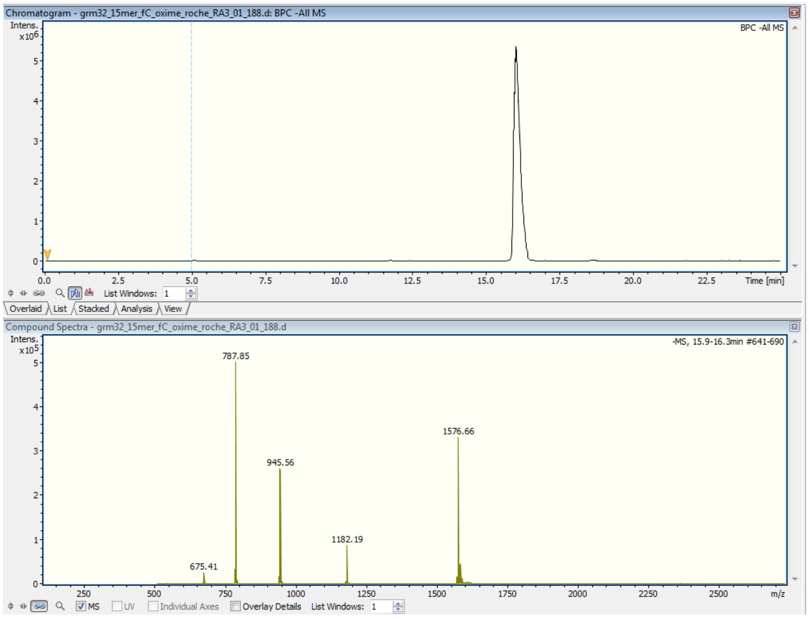

15_5foxC

Oligomer sequences used for qPCR study

Mod_1
5'-GGA GAC TCA GAC AGC GAG CGT TTA AAT AAA TTA AAT AAT ATT AAT ATA TCG ATT AAT AAT AAA TAA TAA TTA ATT AAT ATT CCG TTG ACC TTA CGA TGT CAG G-3'

Mod_4
5'-GGA GAC TCA GAC AGC GAG CGT CGA ATT TCG AAA TAC GAT TAA TAT ATC GAT TAA TAA TAA TAA TTA ATT AAT ATT CCG TTG ACC TTA CGA TGT CAG G-3'

Mod_10
5'-GGA GAC TCA GAC AGC GAG CGT CGA ATT TCG AAC GAC GAT TAA TCG ATC GAT TAA TCG TAA CGA TTA CGT AAC GTT CCG TTG ACC TTA CGA TGT CAG G-3'

qPCR was perfomed on a CFX96 Touch Real-Time PCR Detection System. Bases shown underlined indicate sites of modification.

Oligomer sequences used for polymerase stop assay

Mod_dense_5
`5'-CTC ACC CAC AAC CAC AAA CAA TTT AAA TAT GAT TAA ATA ATA TTA ATA TAC GCG CGC GCG AAT AAT AAT TAA TTA ATA TTG GTT GGA TGG TAG ATG GTG-3'`

Bases shown underlined indicate sites of modification.

Polymerase stop assay protocol

Samples containing DNA template (200 ng), 5'-TAMRA primer (500 nM), dNTPs (200 uM) and 2U of DreamTaq polymerase were made up on ice and mixed via gentle pipetting. After an initial 3 minute denaturation at 95 °C, cycles of denaturation (95 °C, 30s), annealing (52 °C, 30s) and extension (72 °C, 30s) were performed. Aliquots were taken after each extension step and quenched with EDTA. Samples were run as per supplier's instructions on a 15 % TBE-Urea polyacrylamide gel and the TAMRA visualized on a Typhoon imager, before Sybr Gold post-staining and subsequent visualization.

Synthetic Protocols and Spectral Data

(2) Ethyl 3-((1,3-dioxolan-2-yl)methoxy)benzoate (Reported WO 2004/018493)

2-bromomethyl-1,3-dioxolane (4 mL, 38.63 mmol) ethyl-3-hydroxybenzoate (1.66 g, 10.00 mmol), potassium carbonate (2.76 g, 20.00 mmol) and potassium iodide (0.83 g, 5.00 mmol) were heated at 120°C in 10 mL of DMF for 18 hours. The reaction was cooled to room temperature and concentrated under reduced pressure. The residue was partitioned between DCM (150 mL) and water (150 mL). The organic layer was separated and the aqueous layer extracted with DCM (2 x 80 mL). The organic extracts were combined, washed with brine (50 mL), dried ($MgSO_4$) and concentrated under reduced pressure. The residue was purified by column chromatography, eluting with 20 % hexane in DCM. The product was obtained as a colourless oil (2.238 g, 8.90 mmol, 89 %).

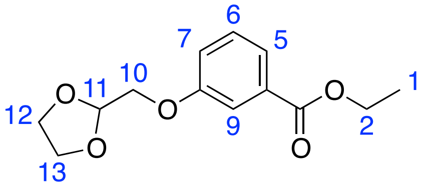

^{1}H NMR (400 MHz, Chloroform-d) δ 7.65 (ddd, J = 7.7, 1.5, 1.3 Hz, 1H, Ar C(5)H), 7.59 (dd, J = 2.7, 1.5 Hz, 1H, Ar C(9)H), 7.33 (dd, J = 7.7, 8.0 Hz, 1H, Ar C(6)H), 7.13 (ddd, J = 8.0, 2.7, 1.3 Hz, 1H, Ar C(7)H), 5.31 (t, J = 4.0 Hz, 1H, C(11)H), 4.36 (q, J = 7.1 Hz, 2H, C(2)H_2), 4.08 (d, J = 4.0 Hz, 2H, ArOC(10)H_2), 4.07 – 3.93 (m, 4H, OCH_2CH_2O), 1.38 (t, J = 7.1 Hz, 3H, C(1)H_3).

(3) Ethyl 3-(2-azido-2-hydroxyethoxy)benzoate

To a mixture of ethyl 3-((1,3-dioxolan-2-yl)methoxy)benzoate (2.2 g, 8.72 mmol) and azidotrimethylsilane (1.26 mL, 9.60 mmol) was added 65µL of $SnCl_4$ at room temperature under N_2. After 2 hours stirring at room temperature 2 % aqueous methanol was added to the reaction mixture before a further 30 minutes stirring. The reaction mixture was concentrated under reduced pressure and then co-evaporated with ethanol (2 x 10mL). The residue was purified by column chromatography (0–1 % methanol in DCM) yielding ethyl 3-(2-azido-2-(2-hydroxyethoxy)ethoxy)benzoate (0.98 g, 39 %) (major) and the title compound as a colourless oil (288 mg, 13 %).

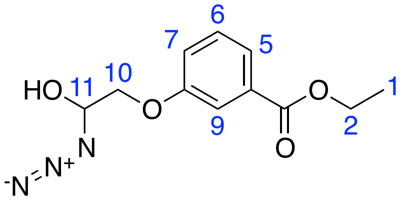

^{1}H NMR (400 MHz, Chloroform-d) δ 7.71 (ddd, *J* = 7.8, 1.5, 1.3 Hz, 1H, Ar C(5)H), 7.57 (dd, *J* = 2.7, 1.5 Hz, 1H, Ar C(9)H), 7.37 (dd, *J* = 8.0, 7.8 Hz, 1H, Ar C(6)H), 7.13 (ddd, *J* = 8.0, 2.7, 1.3 Hz, 1H, Ar C(7)H), 5.06 (t, *J* = 5.3 Hz, 1H, C(11)H), 4.38 (q, *J* = 7.1 Hz, 2H, C(2)H_2), 4.14 (d, *J* = 5.3 Hz, 2H, ArOC(10)H_2), 1.40 (t, *J* = 7.1 Hz, 3H, C(1)H_3).

Ethyl 3-(2-azido-2-((methylsulfonyl)oxy)ethoxy)benzoate

To a stirred solution of ethyl 3-(2-azido-2-hydroxyethoxy)benzoate (150 mg, 0.60 mmol) in DCM (4 mL) and triethylamine (250 µL, 1.80 mmol) in an ice bath under argon was added methanesulfonyl chloride (55 µL, 0.72 mmol) dropwise. After 45 minutes stirring at 0°C the mixture was washed with ice-water (10 mL), 10 % HCl (10 mL) and saturated aqueous $NaHCO_3$ (10 mL). The organic layer was dried ($MgSO_4$) and concentrated under reduced pressure to give the product (197 mg, quant.)

Chemical Formula: $C_{12}H_{15}N_3O_6S$
Molecular Weight: 329.3290

(4) Ethyl 3-(2-azido-2-(((*tert*-butoxycarbonyl)amino)oxy)ethoxy) benzoate

To a stirred solution of DBU (136 µL, 0.9 mmol) and tert-butyl hydroxycarbamate (111 mg, 0.84 mmol) in anhydrous Et_2O (2 mL) cooled in an ice bath under argon, was added a solution of Ethyl 3-(2-azido-2-((methylsulfonyl)oxy)ethoxy)benzoate (197 mg, 0.60 mmol) in Et_2O (2 mL). The mixture was stirred at room temperature for 24 hours, then concentrated and stirred for a further 24 hours. The mixture was then diluted with Et_2O:EtOAC (1:1, 20 mL), washed with aqueous 2 M NH_4Cl (8 mL), then 20 % aqueous NaCl (2 x 8 mL), dried ($MgSO_4$) and concentrated under reduced pressure. Column chromatography (30 % EtOAc in hexane) yielded the product as a colourless oil (120 mg, 0.33 mmol, 55 %). ($C_{16}H_{22}N_4O_6$ requires 366.3691).

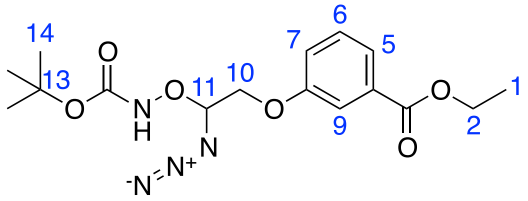

^{1}H NMR (400 MHz, Chloroform-d) δ 7.69 (ddd, *J* = 7.7, 1.5, 1.2 Hz, 1H, Ar C(5)H), 7.57 (dd, *J* = 2.7, 1.5 Hz, 1H, Ar C(9)H), 7.36 (dd, 1*J* = 8.2, 7.7 H, Ar C(6)H), 7.34 (s, 1H, ONHBoc), 7.13 (ddd, *J* = 8.2, 2.7, 1.2 Hz, 1H, Ar C(7)H), 5.35 (t, *J* = 5.1 Hz, 1H, C(11)H), 4.37 (q, *J* = 7.1 Hz, 2H, C(2)H_2), 4.24 (dd, *J* = 10.4, 5.1 Hz, 1H, C(10)H_a), 4.11 (dd, *J* = 10.4, 5.1 Hz, 1H, C(10)H_b), 1.51 (s, 9H, 3xC(14)H_3), 1.39 (t, *J* = 7.1 Hz, 3H, C(1)H_3).

3-(2-azido-2-(((*tert*-butoxycarbonyl)amino)oxy)ethoxy)benzoic acid

To a solution of ethyl 3-(2-azido-2-(((*tert*-butoxycarbonyl)amino)oxy)ethoxy)benzoate (110 mg, 0.30 mmol) in EtOH (4 mL) was added 1 M NaOH (2 mL) and the reaction stirred overnight. The mixture was concentrated under reduced pressure, and then dissolved in water before treatment with 1 M HCl (8 mL). The solution was extracted with EtOAc (20 mL), washed with water (5 mL) and brine (5 mL), dried ($MgSO_4$) and concentrated under reduced pressure. The product is obtained as a colourless oil (98 mg, 0.29 mmol, 97 %).

Chemical Formula: $C_{14}H_{18}N_4O_6$
Molecular Weight: 338.3159

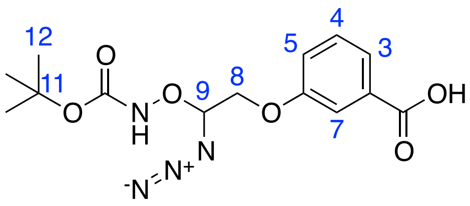

^{1}H NMR (400 MHz, Chloroform-d) δ 7.75 (ddd, *J* = 7.7, 1.5, 1.0 Hz, 1H, Ar C(3)H), 7.63 (dd, *J* = 2.8, 1.5 Hz, 1H, Ar C(7)H), 7.48 (s, 1H, ONHBoc), 7.40 (dd, *J* = 8.3, 7.7 Hz, 1H, Ar C(4)H), 7.19 (ddd, *J* = 8.3, 2.8, 1.0 Hz, 1H, Ar C(5)H), 5.36 (dd, *J* = 5.1, 5.1 Hz, 1H, C(9)H), 4.26 (dd, *J* = 10.4, 5.1 Hz, 1H, C(8)H_a), 4.12 (dd, *J* = 10.4, 5.1 Hz, 2H, C(8)H_b), 1.52 (s, 9H, 3xC(12)H_3).

(5) Perfluorophenyl 3-(2-azido-2-(((*tert*-butoxycarbonyl)amino)oxy)ethoxy)benzoate

To a solution of 3-(2-azido-2-(((*tert*-butoxycarbonyl)amino)oxy)ethoxy)benzoic acid (95 mg, 0.28 mmol) and triethylamine (70 µL, 0.50 mmol) in DMF (4 mL) was added pentafluorophenyl trifluoroacetate (72 µL, 0.42 mmol). The reaction was stirred at room temperature for 30 minutes before concentration under reduced pressure. The crude pentafluorophenyl ester was reacted on without further purification.

Chemical Formula: $C_{20}H_{17}F_5N_4O_6$

Molecular Weight: 504.3642

Biotin-2-aminoethane amide *(N*-(2-aminoethyl)-5-((3a*S*,4*S*,6a*R*)-2-oxohexahydro-1*H*-thieno[3,4-*d*]imidazol-4-yl)pentanamide)

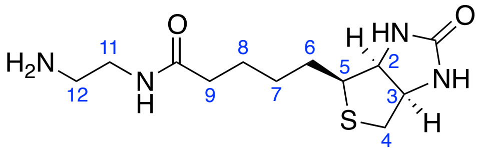

^{1}H NMR (400 MHz, Methanol-d4) δ 4.53 (ddd, *J* = 7.9, 4.9, 1.0 Hz, 1H, C(3)H), 4.34 (dd, *J* = 7.9, 4.5 Hz, 1H, C(2)H), 3.47 (td, *J* = 6.0, 3.0 Hz, 2H, C(11)H_2), 3.24 (ddd, *J* = 8.9, 5.9, 4.5 Hz, 1H, C(5)H), 3.08 (t, *J* = 6.1 Hz, 2H, C(12)H_2), 2.96 (dd, *J* = 12.8, 5.0 Hz, 1H, C(4)H_a), 2.74 (d, *J* = 12.7 Hz, 1H, C(4)H_b), 2.30 (t, 2H, C(9)H_2), 1.87 – 1.56 (m, 4H, C(7)H_2 & C(8)H_2), 1.54 – 1.43 (m, 2H, C(6)H_2).

(6) *Tert*-butyl 1-azido-2-(3-((2-(5-((3a*S*,4*S*,6a*R*)-2-oxohexahydro-1*H*-thieno[3,4-*d*]imidazol-4-yl)pentanamido)ethyl)carbamoyl)phenoxy)ethoxycarbamate

To a solution of the Pfp-ester (141 mg, 0.28 mmol) in DMF (4 mL) cooled to 0°C was slowly added biotin-2-aminoethane amide (240 mg, 0.84 mmol). The reaction mixture was stirred at 0°C for 2 hours under N_2 and then room temperature for a further hour before concentration under reduced pressure. Column chromatography yielded the product as colourless oil (70 mg, 0.12 mmol, 41%).

Chemical Formula: $C_{26}H_{38}N_8O_7S$

Molecular Weight: 606.6943

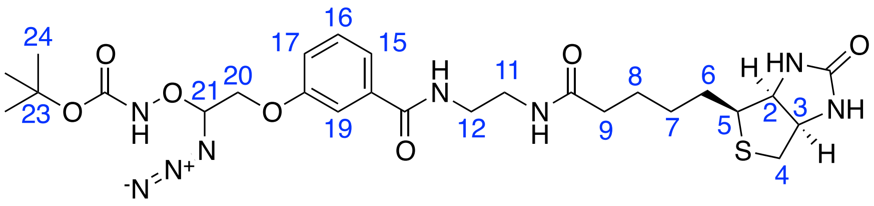

^{1}H NMR (400 MHz, Methanol-d4) δ 8.18 – 8.04 (m, 1H, Ar CH), 7.51 – 7.40 (m, 3H), 7.18 (ddd, J = 8.0, 2.5, 1.2 Hz, 1H, Ar CH), 5.35 (dd, J = 5.0, 4.9 Hz, 1H, C(21)H), 4.55 – 4.44 (m, 1H, C(3)H), 4.33 – 4.21 (m, 2H, C(2)H & C(20)H_a), 4.16 (dd, J = 10.6, 5.0 Hz, 1H. C(20)H_b), 3.56-3.52 (m, 2H, CONHCH_2), 3.51 – 3.43 (m, 2H, CONHCH_2), 3.18 – 3.06 (m, 1H, C(5)H), 2.91 (dd, J = 12.7, 4.9 Hz, 1H, C(4)H_a), 2.71 (d, J = 12.7 Hz, 1H, C(4)H_b), 2.25 (t, 2H, C(9)H_2), 1.78 – 1.62 (m, 4H, CH_2, C(7)H_2 & C(8)H_2), 1.53 (s, 9H, 3xC(24)H_3), 1.43 (dt, J = 7.8, 7.7 Hz, 2H, C(6)H_2).

(1) 3-(2-(aminooxy)-2-azidoethoxy)-*N*-(2-(5-((3a*S*,4*S*,6a*R*)-2-oxohexahydro-1*H*-thieno[3,4-*d*]imidazol-4-yl)pentanamido)ethyl)benzamide

The boc-protected species (22 mg, 0.04 mmol) was dissolved in 1.25 M HCl:MeOH (5 mL) solution and stirred at 40 °C for 90 minutes. The crude product was purified by HPLC (0 – 100% ACN in water, retention time 14.2 minutes). Product obtained as a white powder following lyophilization (18 mg, quant.). HRMS m/z found 507.2150 ($C_{21}H_{31}N_8O_5S^+$ requires 507.2138, +2.4 ppm).

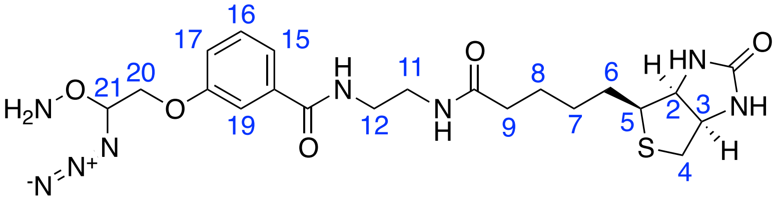

^{1}H NMR (500 MHz, DMSO-d6) δ 8.48 (t, *J* = 5.5 Hz, 1H, CONH), 7.92 (t, *J* = 5.7 Hz, 1H, CONH), 7.49 – 7.34 (m, 3H, Ar CH), 7.11 (ddd, *J* = 8.1, 2.6, 1.0 Hz, 1H, C(17)H), 6.40 (s br, 1H, NHCONH), 6.35 (s br, 1H, NHCONH), 5.28 (dd, *J* = 5.5, 4.5 Hz, 1H, C(21)H), 4.31 – 4.26 (ddd, *J* = 8.0, 5.1, 1 Hz, 1H, C(3)H), 4.14 – 4.04 (m, 3H, C(20)H_aH_b and C(2)H), 3.29 (dt, *J* = 6.6, 5.9 Hz, 2H, C(11)H_2), 3.21 (dt, *J* = 7.2, 6.6 Hz, 2H, C(12)H_2), 3.05 (ddd, *J* = 8.5, 6.3, 4.4 Hz, 1H, C(5)H), 2.80 (dd, *J* = 12.4, 5.1 Hz, 1H, C(4)H_a), 2.57 (d, *J* = 12.4 Hz, 1H, C(4)H_b), 2.06 (t, *J* = 7.4 Hz, 2H, C(9)H_2), 1.58 (ddt, *J* = 14.5, 12.2, 4.3 Hz, 1H), 1.54 – 1.38 (m, 3H, CH_2), 1.28 (m, 2H, CH_2).

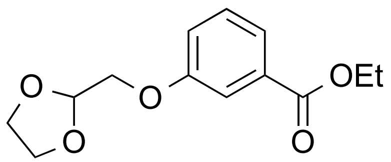

2

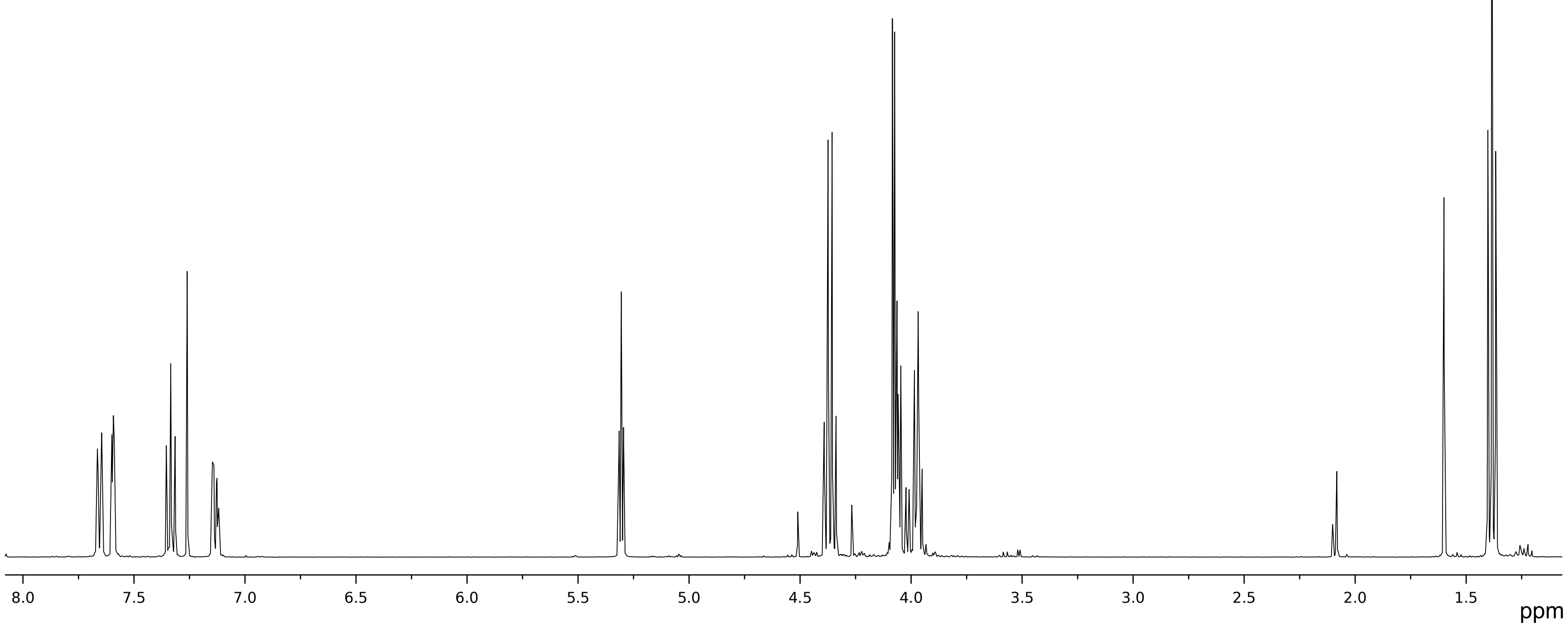

^{1}H NMR (400 MHz, $CDCl_3$) of *Molecule 2*

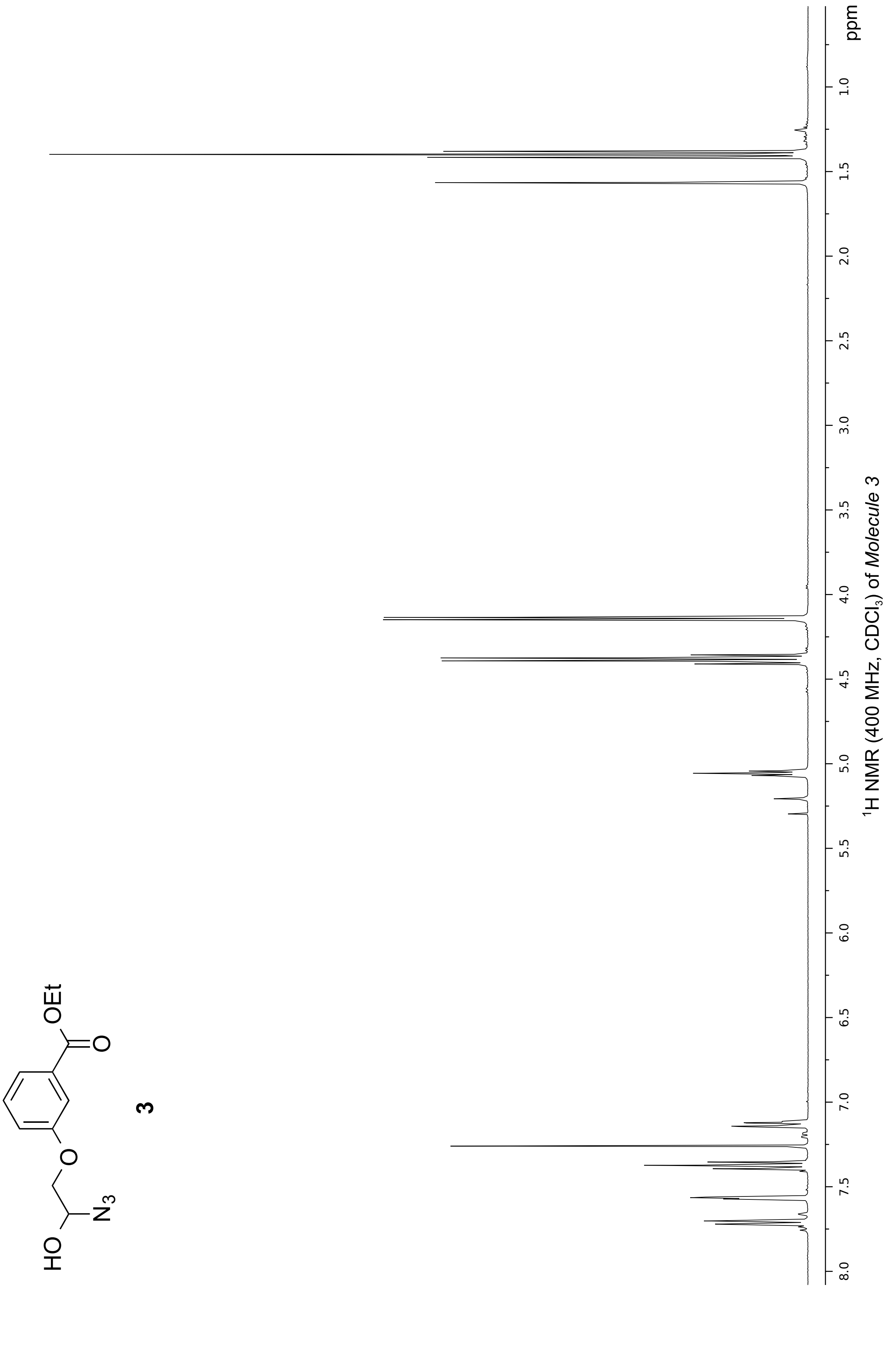

^{1}H NMR (400 MHz, $CDCl_3$) of *Molecule 3*

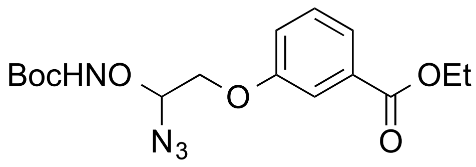

4

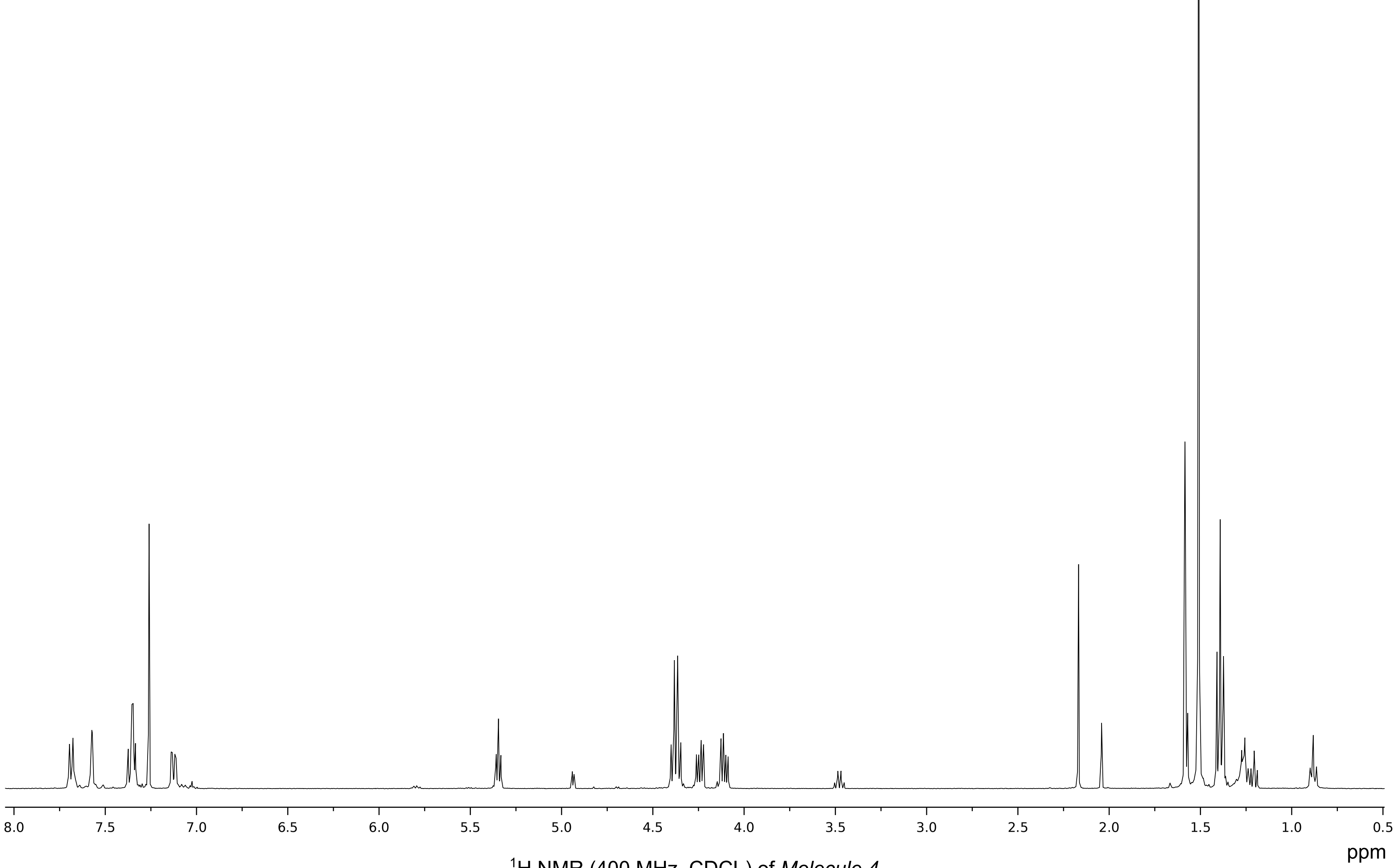

^{1}H NMR (400 MHz, $CDCl_3$) of *Molecule 4*

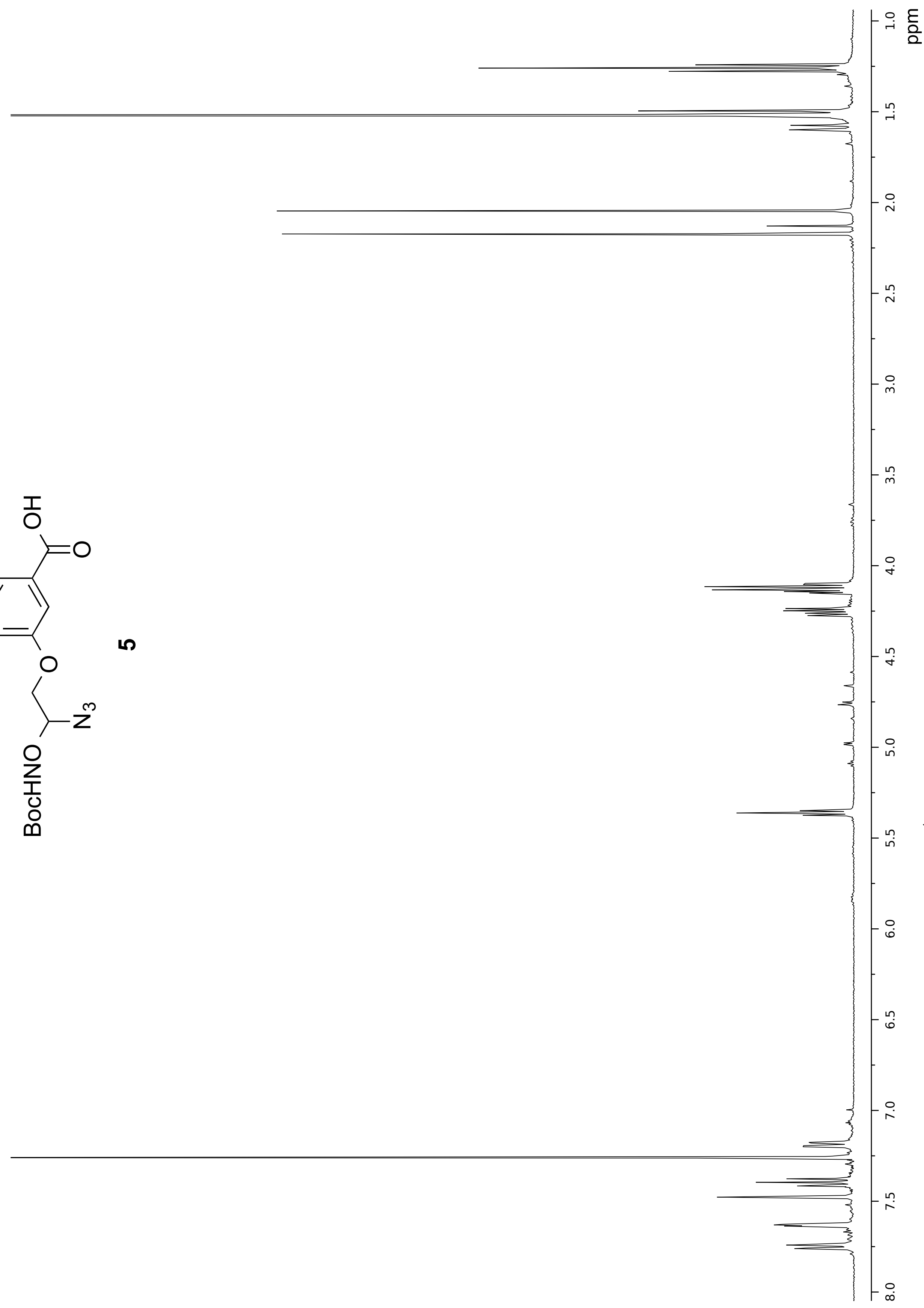

^{1}H NMR (400 MHz, $CDCl_3$) of *Molecule 5*

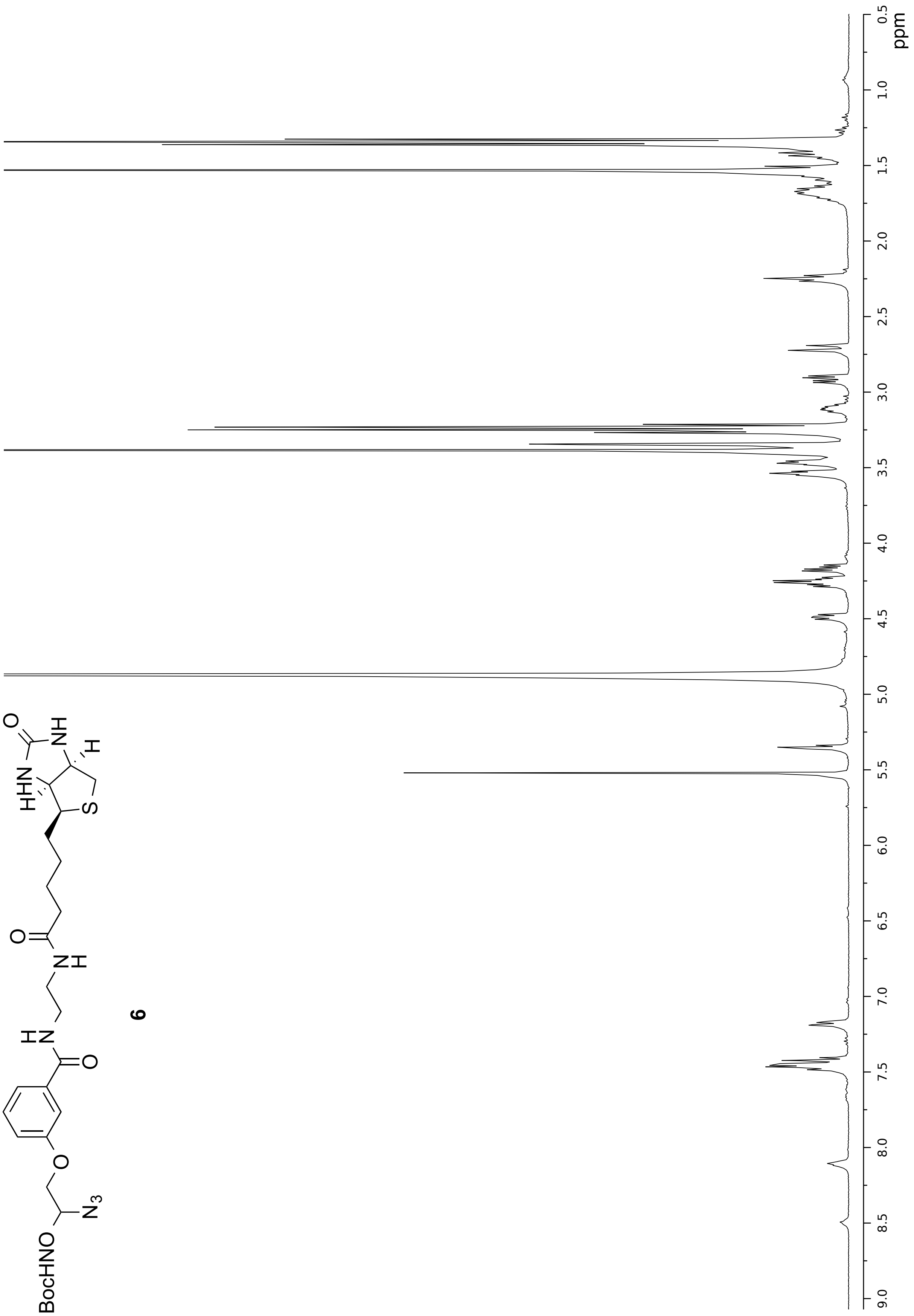

^{1}H NMR (400 MHz, MeOD) of *Molecule* 6

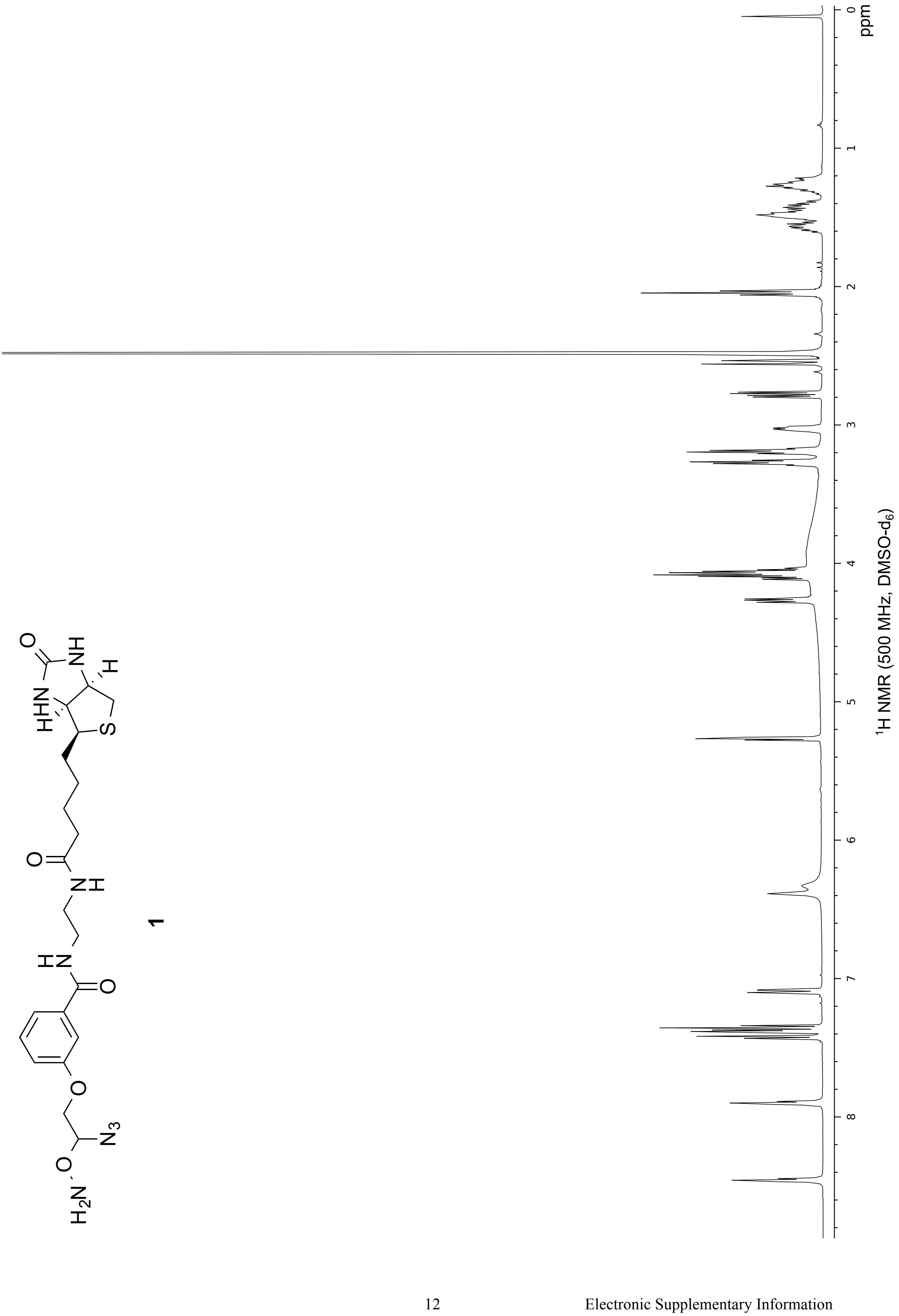
1
ppm
^{1}H NMR (500 MHz, DMSO-d$_6$)
